# Effect of public corruption on the COVID-19 immunization progress

**DOI:** 10.1038/s41598-021-02802-1

**Published:** 2021-12-06

**Authors:** Mohammad Reza Farzanegan, Hans Philipp Hofmann

**Affiliations:** 1 Economics of the Middle East Research Group & School of Business and Economics, Center for Near and Middle Eastern Studies (CNMS), Philipps-Universität Marburg, Deutschhausstr. 12, 35037 Marburg, Germany; 2 CESifo, Munich, Germany; 3 ERF, Cairo, Egypt

**Keywords:** Diseases, Health care, Risk factors

## Abstract

The coronavirus disease (COVID-19) outbreak has resulted in the death of over four million people since late 2019. To reduce the human and economic costs of COVID-19, different vaccines have been developed and distributed across countries. There has been significant cross-country variation in the vaccination of people against COVID-19. In this study, we focus on public corruption to explain the significant cause of cross-country variation in immunization progress. We suggest that countries with a higher degree of public corruption have been less successful in the vaccination of their population, controlling for other important determinants of immunization progress.

## Introduction

The end of 2019 saw the emergence of a novel and particularly infectious disease which had a sudden and profound global impact. The new coronavirus spread rapidly and soon took over the planet. There have been more than 213 million confirmed cases and close to 4.5 million deaths as of August 2021. To limit the spread of the virus, COVID-19 containment measures were implemented by countries. These measures, such as social distancing, entail economic and psychological costs (see for example^1–3^). The only long-term response to the crisis is the immunization against the severe acute respiratory syndrome coronavirus type 2 (SARS-CoV-2).

Several vaccines have been developed and offer high protection against disease and infection from the virus. For example, the Pfizer/BioNTech vaccine has 92% efficacy at preventing the COVID-19 disease for the ancestral type and Alpha variant and 90% for the Beta, Gamma, and Delta variants. The vaccine has 86% efficacy at preventing the infection for the ancestral and Alpha and has 78% efficacy for the Beta, Gamma, and Delta variants^4^. Countries such as Bhutan, Israel and the Seychelles have already vaccinated large contingents of their adult population^5^. However, there have been challenges in vaccinating the global population. The problem of corruption in the vaccine process has gained the attention of media and international organizations.

In the report “COVID-19 Vaccines and Corruption Risks: Preventing Corruption in the Manufacture, Allocation and Distribution of Vaccines,” the United Nations Office on Drugs and Crime^6^ discusses the potential risks of corruption in the distribution process, where vaccine doses could be stolen, the theft of emergency funding and opportunities for nepotism, favoritism, and corrupted procurement systems. Transparency International^7^ and the European Anti-Fraud Office^8^ raised similar concerns regarding the distribution of vaccines.

The supply shortage of COVID-19 vaccines creates opportunities for corruption throughout the world. In South America, Peruvian and Argentinian politicians and their families received vaccinations prior to being officially eligible for them^9^. There have also been reports of wide-ranging corruption scandals related to the COVID-19 vaccine in Brazil and Venezuela^9^. In Spain, local mayors received preferential access to vaccine doses before they were widely available to the general public^10,11^. In Italy, there have been reports of vaccine sales on the grey market^12,13^. Corruption scandals around the vaccine were also reported in Lebanon, South Africa and China^14,15^. Another example is Iran, which is struggling with significant mortality rates due to COVID-19 and slow vaccination progress. In May 2021, Kianoush Jahanpur, the spokesman for Iran's Food and Drug Administration, noted significant over-invoicing of imported vaccines by private firms (up to 12 times higher than the real price of the vaccines). Such firms with access to subsidized rates on vaccines are incentivized to over-invoice their imports and sell the additional amounts in the black market for foreign exchange at a high premium^16^. In addition to the recent COVID-19 vaccine scandals, it is worth mentioning that some of the pharmaceutical companies manufacturing the COVID-19 vaccine (AstraZeneca, Johnson & Johnson, Pfizer) were sued over bribery prior to the pandemic^15^. These examples argue for the possible destructive effects of public corruption on the successful vaccination against COVID-19. However, a data-driven cross-country investigation on the role of corruption in vaccinations is lacking. In this study, using data on the share of the population who have been vaccinated against COVID-19, public corruption, healthcare capabilities and economic indicators from over 90 countries, we examine the association between corruption and COVID-19 vaccination coverage. We find strong evidence of the damaging effects of pre-pandemic corruption on immunization progress across countries.

## Literature review

Lambsdorff^17^ defines corruption as “[…] the misuse of public power for private benefit.” Therefore, corruption occurs at the intersection of public spending and the private sector. Misuse is defined as a behavior that deviates from the formal and informal rules of a political society or “[…] more generally, where narrow interests are followed at the expense of the broader interests of the public at large”^17^. Corruption can take different forms such as extortion, fraud, embezzlement and bribery^17^.

But how can corruption affect the speed and scale of worldwide vaccination projects? In light of the extraordinary speed and global scale of COVID-19 vaccine distribution, Goel et al.^18^ identify, based on pre-pandemic research on corruption, at which stages of the vaccination process can public power be misused for private benefit. Goel et al. argue that the urgent political need for a vaccine creates an additional layer of government intervention compared to previous distribution processes of pharmaceutical goods. The accompanying increased number of involved bureaucrats could create opportunities for corrupt activities. Hastily negotiated contracts could give incentives for rent-extracting behavior. Goel et al. explain that at the vaccine development and approval stages, pharmaceutical companies might bribe regulatory institutions to expedite approvals of new vaccines. Finally, they suggest that in the distribution process, consumers might try to bribe bureaucrats for two reasons: to obtain vaccines before their official eligibility and to seek additional doses to subsequently sell on the grey market.

Another channel for the effect of corruption on vaccination progress is the quality of health systems. The quality of the health sector essentially defines the speed and effectiveness of the COVID-19 vaccination process. However, the health sector is also prone to corruption. Savedoff and Hussmann^19^ state: “No other sector has the specific mix of uncertainty, asymmetric information and large numbers of dispersed actors that characterize the health sector. As a result, susceptibility to corruption is a systemic feature of health systems […].” The authors underscore the enormous amount of global government spending on health, which would make the health sector highly profitable and therefore especially vulnerable to corruption. Transparency International estimates that 7.5 trillion dollars every year are spent on public health globally, 500 billion dollars of which is misappropriated due to corruption^20^.

Sommer^21^ argues that corruption in the health sector reduces the available capital for health, increases the costs for patients, inhibits service improvement, reduces the quality of care and generally hinders reform and improvement. Countries with health sectors that suffered from corruption prior to the crisis could be less effective during the COVID-19 pandemic. Farzanegan^22^, for example, has shown that the level of corruption is positively and significantly associated with COVID-19 fatality rates across countries. The “Nikkei COVID-19 Recovery Index,” ranks the vaccination progress, country-level infection management and social mobility for more than 120 countries. 14 out of 15 countries at the bottom of the ranking are the worst performing countries in the fight against the pandemic in September 2021 and are negative in the control of corruption index from the World Governance Indicators, showing higher degrees of corruption. Conversely, 13 out of the 15 top performing countries according to the index are in positive in the corruption index^23^. The nexus between corruption and governmental and health sector performance during the pandemic could also affect the speed and scale of vaccination projects worldwide.

Moreover, there is empirical evidence for the substantial negative effects of corruption on the health sector. Hanf et al.^24^ find a positive relationship between corruption and child mortality. The authors estimate that more than 140,000 deaths of children every year could be indirectly attributed to corruption. Sommer^21^ also focuses on child and infant mortality as the dependent variable and concludes that the interaction between corruption and health expenditures negatively affects child and infant mortality. Li et al.^25^ verify the negative effects of corruption on five different measures of health outcomes and conclude that corruption causes deaths. Habibov^26^ finds a negative relationship between corruption experienced in the healthcare sector and satisfaction with healthcare as subjective indicators. Achim et al.^27^ find that higher levels of corruption negatively affect physical (expressed as life expectancy and mortality) and mental health (expressed by happiness). Azfar and Gurgur^28^ find that in areas in the Philippines with widespread corruption, respondents reported longer wait times in public clinics and a higher frequency of being denied vaccines even prior to the COVID-19 crisis. Among other health outcomes, which were negatively associated with corruption, the authors find that corruption reduces immunization rates and delays the vaccination of newborns. Factor and Kang^29^ analyze the effects of corruption on different health indicators, including immunization. Their results show that higher corruption is associated with lower levels of health expenditures as a percentage of GDP per capita, and with poorer health outcomes in general. Higher levels of corruption show a negative effect particularly on Diphtheria and tetanus toxoids and pertussis (DPT) immunization rates.

Another channel through which corruption may affect COVID-19 vaccination progress is social and political trust. Studies find that corruption negatively affects trust (see examples^30–32^). Low levels of political trust may decrease the acceptance of vaccines against the COVID-19 virus. Empirical research finds a positive effect of trust on vaccine acceptance during previous immunization campaigns^33,34^. During COVID-19, trust in institutions and the health sector is especially crucial due to the shortened clinical trials and emergency approvals of the vaccines in many countries. Lazarus et al.^35^ report that higher levels of trust in information provided by the government are associated with higher COVID-19 vaccine acceptance.

In addition to the direct causes of corruption on the vaccination progress, the development stage of the COVID-19 vaccines needs to be considered. The first vaccine approved by the US Food and Drug Administration was from Pfizer/BioNTech^36^. BioNTech is a German startup that collaborated with Pfizer to develop a COVID-19 vaccine. One of the co-founders of the BioNTech Company, Dr. Ugur Sahin, commented in an interview with the New York Times regarding the development of the vaccine: “There are not too many companies on the planet which have the capacity and the competence to do it so fast as we can do it […]”^37^. The fast development of effective vaccines against the COVID-19 virus presupposed a constructive climate for entrepreneurship and highly educated scientists. Higher levels of corruption have been shown to have negative effects on productive entrepreneurship^38^ and quality of education^39^. Therefore, corruption may additionally have an indirect impact on vaccination projects by reducing entrepreneurship and the quality of education in the long term. Table 1 presents a summary of related studies.

**Table 1:** Review of the literature on the governance-vaccination nexus.

Study	Subject of investigation	Approach	Findings
**Effect of corruption on the health sector**			
Achim et al.^27^	Effect of corruption on population health	Pooled OLS 185 countries (54 high-income and 131 low-income countries) Period of the analysis: 2005–2017	Higher levels of corruption are negatively associated with physical health (expressed as life expectancy and Mortality rate) and mental health (expressed by happiness)
Habibov^26^	Effect of corruption on healthcare satisfaction in transitional countries	OLS regression and two-stage 2SLS regression Use the “Life in Transition Survey” Observations: (N = 8655) in 12 post-socialist countries	Negative relationship between corruption and healthcare satisfaction
Hanf et al.^24^	Effect of corruption on national under-five mortality rate	Multivariate linear regression modelling 178 countries Cross-sectional	Positive effect of corruption on child mortality Authors estimate that more than 140,000 annual children deaths could be indirectly attributed to corruption
Holmberg and Rothstein^40^	Effect of quality of government on population health	Multivariate linear regression > 120 countries cross-sectional	Higher levels of the QoG index are positively associated with life expectancy and higher levels of subjective health feelings Complementary, higher levels of the QoG index were negatively associated with mortality rates for children and mothers
Lio and Lee^41^	Effect of corruption on five indicators for population health	Ordinary least squares (OLS), fixed-effects 119 countries for the period 2005–2011	Lower levels of corruption are positively associated with: Longer life expectancy Lower infant mortality rate Lower under-five mortality rate
Sommer^21^	Investigate the effect of the interaction between health expenditure and corruption in the executive and in the public sectors on infant and child mortality	Two-way fixed effects models 90 lowand middle-income countries timeframe: 1996 to 2012 Panel data	Negative interaction effect of corruption and health expenditure on child and infant mortality
**Effect of corruption on the health sector—focus on immunization**			
Azfar and Gurgur^28^	Effect of corruption on health outcomes	Random effects, tobit, ordinary-least Philippines: Survey data	Corruption reduces immunization rates and delays the vaccination of newborns. One standard deviation (≈10%) increase in corruption reduces the immunization rate by approximately 11–19% Effect of corruption on health outcomes depends on the geography of the regions (rural/urban) Lower-income strata were more affected by corruption
Factor and Kang^29^	Developing a theoretical framework for understanding the impact of corruption. Analyzing the effects of corruption on different health indicators such as immunization	Structural equation models 133 countries	Higher corruption is associated with lower levels of health expenditures as a percentage of GDP per capita, and with poorer health outcomes in general Corruption has negative effect on DPT immunization rates
Goel and Nelson^44^	Examine socio-economic driver of two dependent variables; administration and delivery efficiency of Covid-19 vaccines	OLS regression 50 States of the United States Data was collected for two different periods (12. January and 2. February 2021)	Nursing homes per capita, Covid-19 deaths and amount of health workers are positively associated with the delivery efficiency of Covid-19 vaccines Centralized public health agency is associated with vaccination efficiency Corruption (5-year average) shows positive effect for both dependent variables but no statistical significance for the variable which captures the dissemination of vaccinations in the second time period (2. February 2021)
Li et al.^25^	Investigate the effect of corruption on health outcomes/dependent variables were among others DPT immunization and measles immunization	Ordinary least squares (OLS), fixed-effects and two-stage least squares (2SLS) estimation methods, ≈ 150 countries/cross-country panel data	Negative effect of corruption on health outcomes such as DPT and measles immunization

In short, based on additional government intervention due to the urgent response required by the virus, the high potential to sell vaccines on the grey market, the negative effects of corruption on the health sector and its role in the vaccine distribution process and the potential negative effect of corruption on public trust in the vaccine, we expect corruption to be negatively associated with cross-country COVID-19 vaccination progress. Our hypothesis is therefore:

**Hypothesis** Higher levels of public corruption have a negative effect on the percentage of the population who are partially or fully vaccinated against COVID-19, *ceteris paribus.*

## Methods

### Study variables and data sources

Our main dependent variables are the percentage of the population who received at least their first dose of a COVID-19 vaccine and the percentage of the population who are fully vaccinated, where “fully vaccinated” refers to people who have been fully vaccinated with either a single- or two-dose vaccine. The data for vaccination coverage is from Bloomberg^42^. Our dependent variable shows the status of countries on 30 August 2021.

The primary independent variable is a measure of corruption. We use the control of corruption index from World Governance Indicators^43^, which shows the perception of the extent to which public power is exercised for private gain and capture of the state by private interest and elites. We reverse this index by multiplying it by − 1. Thus, a higher score means a higher level of perceived corruption. We use the scores of countries in 2020 (our results also hold if we use corruption scores from 2019, which may be useful to reduce the risk of reverse feedback from the COVID-19 outbreak in 2020 and 2021 and vaccination projects). Goel and Nelson^44^ explain the implications of COVID-19 vaccine rollouts on corruption in the case study of the US. For a robustness check, we also use the corruption perception index (CPI) from Transparency International in 2020. CPI is a composite index based on a combination of 13 surveys that shows how corrupt a country’s public sector is perceived to be by experts and business executives^45^. Theoretically, it is in the range of 0 (most corrupt) to 100 (least corrupt). We subtract the original scores from 100, thus higher values refer to more corruption in the public sector. There is a significant and positive correlation between the WGI and TI corruption indicators in our sample (r = 0.98).

Progress in COVID-19 immunization is not just a function of administrative corruption. To check for other possible drivers of speed and coverage of vaccination projects, we consider a set of socio-economic, health and institutional indicators. The inclusion of such variables is important because they can be correlated with both corruption and vaccination coverage. These control variables are the logarithm (log) of GDP per capita, the log of physicians (per 1,000 people), the log of domestic general government health expenditure per capita, the log of nurses and midwives (per 1,000 people), and urban population (% of total population). These variables are taken from the World Development Indicators by the World Bank^46^ capture the pre-pandemic socio-economic conditions of countries. Earlier studies have shown a significant correlation between the level of economic development and corruption. A line of literature refers to the possible positive association (greasing the wheels hypothesis) when there is a high regulatory burden in an economy (e.g.,^47,48^) while the most recent studies show a negative association between corruption and income of a nation (sanding the wheels hypothesis) (e.g.,^49,50^). Corruption is also associated with pre-pandemic health infrastructure and resources. It is shown that corruption distorts the allocation of a government’s budget toward areas where bribes can be more easily obtained. In such economies, the areas such as public health and education are under-invested^51–53^. Obviously an equipped and efficient health care system can manage large-scale projects, such as vaccinations, more effectively. Corruption may hinder the successful implementation of vaccination through its distorting effect on the health sector. Finally, we have also controlled for the share of the urban population. It is also seen that higher levels of corruption may destabilize a political system in countries with higher levels of youth urban population^54^. An unstable political system will be less successful in efficiently and effectively implementing major vaccination projects.

In addition, we control for the quality of political institutions by including the Polity2 index, which is taken from Marshall et al.^55^, and the government effectiveness index in 2020 from WGI^43^. Corruption is associated with the type of political regime, which is an important contributor toward successful vaccination projects. The government effectiveness measurement aims to capture perceptions of the quality of public services, the quality of the civil service and the degree of its independence from political pressures. In addition, it shows the quality of policy formulation and implementation and the credibility of the government's commitment to such policies. This dimension of governance is relevant in our analysis because vaccinations are an important public service and needs significant state capacity, coordination and commitment by government agencies. Government effectiveness has already been proven to be negatively associated with COVID-19 mortality^56^ and can be an important transmission channel for the effect of public corruption on vaccination progress. Moreover, countries with high ethnic fractionalization may suffer from a variety of socio-economic and policy distortions^57^. The risk of internal conflict and rent-seeking is higher in countries with higher degrees of ethnic fractionalization^57^. Thus, we also control for this issue using the fractionalization index from Drazanova^58^. Also, countries with more integration in the international markets and more economic openness may have faster access to suppliers of COVID-19 vaccines. Thus, we also control for globalization using the 2018 index (latest available year) from Gygli et al.^59^ The issue of international contacts was also examined by Farzanegan et al.^60^ during the COVID-19 outbreaks across countries. Finally, we control for continent dummy variables that consider other regional characteristics which may explain cross-country variations in the progress of vaccination against COVID-19.

Supplementary Information 1 provides detailed information on the variables and their sources. Table 2 presents the summary statistics of variables.

**Table 2:** Descriptive statistics.

Variable	Obs	Mean	SD	Min	Max	Sources
Given 1 dose (30 Aug. 2021) % of population	190	35.95	26.46	0.10	83.60	Bloomberg^42^
Fully vaccinated (30 Aug. 2021) % of population	186	28.45	24.81	0.10	83.40	Bloomberg^42^
Control of corruption (in 2020) (re-scaled)*	188	− 0.01	1.01	− 2.27	1.91	WGI^43^
Corruption perceptions index (in 2020) (re-scaled)**	171	55.95	18.95	12	88	Transparency International^45^
Log of GDP per capita (2017–2019)	183	8.81	1.47	5.94	12.19	WDI^46^
Log of physicians (per 1,000 people) (2017–2019)	113	0.09	1.33	− 3.33	2.12	WDI^46^
Log of domestic general government health expenditure per capita (2017–2019)	177	5.84	1.74	1.42	8.65	WDI^46^
Log of nurses and midwives (per 1000 people) (2017–2019)	146	0.98	1.17	− 2.64	4.54	WDI^46^
Urban population (% of population) (2017–2019)	191	60.35	23.93	13.17	100	WDI^46^
Polity2 index (in 2018)	160	4.38	6.04	− 10	10	Marshall et al^55^
Government effectiveness (in 2020)	188	0.03	1.00	− 2.34	2.34	WGI^43^
Fractionalization	149	0.46	0.25	0.02	0.89	Drazanova^58^
Globalization (in 2018)	184	62.78	14.24	30.16	90.79	Gygli et al.^59^

*Re-scaled by multiplying the original index by − 1. Higher scores show higher levels of petty and grand corruption.

**Rescaled by subtracting the original index from 100. Higher scores show higher levels of public corruption.

The baseline econometric model has the following form and we estimate it with ordinary least squares (OLS) and robust standard errors.

1 $$ covid19\_vaccinations_{i} = \alpha + \beta_{1} \cdot corruption_{i} + \beta ^{\prime}_{2} \cdot Z_{i} + \varepsilon_{i} $$

The subscript *i* refers to country *i*. Corruption denotes the corruption index. In our baseline specification, we use the corruption index from 2020 and expect a negative influence of corruption on the percentage of the population who have been vaccinated against COVID-19. *Z* is a vector of control variables including continent dummy variables. We distinguish between six different continents: Africa, Asia, Europe, North America, South America, and Oceania (reference category). In addition, *Z* contains the political-socio-economic control variables (in most cases, to increase the number of observations, the average values between 2017 and 2019 are used).

## Results

Table 3 shows the estimation results for the share of people in the total population who were vaccinated once as the dependent variable. Table 4 shows the share of people who are fully vaccinated as the dependent variable. Our focus lies on corruption as the main explanatory variable. The first 13 models show the level of corruption according to the (reversed) control of corruption index from the World Governance Indicators. Model 14 in Tables 3 and 4 includes a robustness test by applying the level of corruption in countries according to the (modified) corruption perceptions index from Transparency International. Higher levels in the index can be understood as higher levels of public corruption.

**Table 3:** Regression results with the % of the population who have received one COVID-19 vaccine as of 30.08.2021and corruption as the main explanatory variable.

	(1)	(2)	(3)	(4)	(5)	(6)	(7)	(8)	(9)	(10)	(11)	(12)	(13)	(14)
**Given 1 dose (by 30 August 2021)**														
Corruption in 2020 (WGI)	− 18.391*** (− 17.45)	− 14.678*** (− 10.80)	− 7.146*** (− 3.87)	− 12.716*** (− 6.74)	− 7.959*** (− 4.40)	− 10.203*** (− 5.60)	− 12.138*** (− 7.98)	− 17.008*** (− 13.78)	− 1.506 (− 0.44)	− 16.037*** (− 12.36)	− 11.716*** (− 6.62)	− 11.409** (− 2.32)	− 10.422*** (− 3.08)	
Corruption in 2020 (TI)														− 0.541*** (− 2.93)
Log of GDP per capita			8.868*** (6.55)									5.304 (1.61)	8.105*** (2.91)	8.435*** (2.97)
Log of physicians (per 1,000 people)				5.575*** (3.45)								0.144 (0.05)	1.238 (0.49)	0.655 (0.26)
Log of government health expenditure					7.163*** (5.52)							3.969 (1.20)		
Log of nurses and midwives						4.851*** (3.36)						− 1.650 (− 0.71)	− 0.844 (− 0.42)	− 0.828 (− 0.42)
Urban population (% of total population)							0.272*** (4.53)					− 0.113 (− 0.79)	− 0.075 (− 0.56)	− 0.086 (− 0.62)
Polity2 index								− 0.690** (− 2.36)				− 0.703** (− 2.46)	− 0.733** (− 2.59)	− 0.629** (− 2.08)
Government effectiveness									15.889*** (4.67)			− 2.120 (− 0.35)		
Fractionalization										− 3.201 (− 0.55)		6.384 (0.95)	4.850 (0.73)	4.147 (0.62)
Globalization											0.576*** (3.61)	0.375 (0.92)	0.260 (0.70)	0.228 (0.56)
Africa		− 10.407 (− 1.56)	− 6.874 (− 1.12)	9.319 (1.50)	− 5.821 (− 0.89)	− 10.992* (− 1.68)	− 13.221** (− 2.00)	− 3.002 (− 0.45)	− 8.970 (− 1.59)	− 1.370 (− 0.13)	− 7.227 (− 1.04)	14.691 (1.65)	15.277* (1.80)	14.896* (1.72)
Asia		11.293* (1.69)	7.009 (1.11)	21.261*** (3.43)	7.734 (1.17)	10.076 (1.49)	5.700 (0.84)	17.736** (2.53)	5.901 (0.98)	21.523** (2.10)	10.321 (1.43)	28.245*** (3.85)	27.886*** (3.86)	28.022*** (3.68)
North America		10.703 (1.49)	3.368 (0.49)	16.046*** (2.63)	1.078 (0.15)	6.353 (0.87)	5.912 (0.85)	27.426*** (3.62)	7.030 (1.15)	26.530** (2.46)	11.330 (1.46)	28.881*** (4.04)	30.612*** (4.58)	31.132*** (4.36)
South America		21.291*** (3.13)	16.084** (2.41)	32.862*** (5.92)	12.133* (1.72)	19.674*** (2.97)	11.882* (1.67)	31.386*** (4.62)	19.984*** (3.29)	31.673*** (3.11)	19.429*** (2.64)	43.552*** (6.80)	44.785*** (7.78)	45.534*** (7.40)
Europe		12.947** (2.04)	4.114 (0.66)	23.504*** (4.56)	3.143 (0.47)	11.881* (1.78)	6.825 (1.03)	21.654*** (3.37)	7.225 (1.29)	20.179** (2.09)	3.895 (0.50)	25.124*** (3.84)	26.578*** (4.16)	27.378*** (3.92)
Obervations	183	183	179	112	174	145	182	158	183	147	179	92	93	93
R-squared	0.50	0.64	0.71	0.72	0.70	0.65	0.68	0.72	0.69	0.70	0.68	0.82	0.81	0.81

Method of estimation is ordinary lease squares. Robust *t* statistics are in parentheses.

***, **, *Statistical significance at the 1%, 5%, and 10% levels, respectively.

**Table 4:** Regression results with the % of the population who have been fully vaccinated against COVID-19 as of 30.08.2021 and corruption as the main explanatory variable.

	(1)	(2)	(3)	(4)	(5)	(6)	(7)	(8)	(9)	(10)	(11)	(12)	(13)	(14)
**Fully vaccinated (by 30 August 2021)**														
Corruption in 2020 (WGI)	− 16.836*** (− 14.89)	− 13.267*** (− 9.07)	− 5.724*** (− 2.78)	− 11.592*** (− 5.62)	− 7.402*** (− 3.71)	− 9.193*** (− 4.21)	− 10.938*** (− 6.57)	− 14.930*** (− 10.71)	− 0.763 (− 0.22)	− 14.500*** (− 10.30)	− 11.358*** (− 6.09)	− 9.037 (− 1.60)	− 10.102*** (− 2.75)	
Corruption in 2020 (TI)														− 0.514** (− 2.52)
Log of GDP per capita			8.665*** (5.60)									6.142* (1.98)	6.850** (2.59)	7.253*** (2.71)
Log of physicians (per 1,000 people)				4.298** (2.57)								1.070 (0.39)	1.199 (0.47)	0.650 (0.26)
Log of government health expenditure					5.824*** (4.23)							0.717 (0.23)		
Log of nurses and midwives						3.224* (1.89)						− 1.927 (− 0.82)	− 1.873 (− 0.83)	− 1.783 (− 0.80)
Urban population (% of total population)							0.239*** (3.69)					− 0.046 (− 0.35)	− 0.054 (− 0.43)	− 0.068 (− 0.53)
Polity2 index								− 0.540* (− 1.80)				− 0.604* (− 1.93)	− 0.616* (− 1.97)	− 0.526 (− 1.58)
Government effectiveness									15.084*** (4.28)			1.617 (0.23)		
Fractionalization										− 1.272 (− 0.24)		6.054 (0.90)	6.133 (0.98)	5.303 (0.84)
Globalization											0.411** (2.48)	0.230 (0.54)	0.284 (0.80)	0.255 (0.65)
Africa		− 1.248 (− 0.17)	2.936 (0.47)	15.342* (1.84)	3.275 (0.48)	− 2.857 (− 0.41)	− 2.629 (− 0.37)	6.488 (1.44)	1.104 (0.18)	11.478* (1.88)	5.572 (0.96)	25.284*** (2.90)	25.335*** (2.94)	24.856*** (2.82)
Asia		13.362* (1.85)	10.195 (1.55)	21.520** (2.62)	11.296 (1.58)	12.461* (1.67)	9.579 (1.29)	20.849*** (4.25)	9.162 (1.40)	28.245*** (4.64)	17.260*** (2.85)	32.690*** (4.42)	32.955*** (4.46)	32.960*** (4.25)
North America		13.518* (1.81)	7.471 (1.10)	17.462** (2.27)	5.391 (0.74)	8.555 (1.15)	10.460 (1.41)	25.747*** (4.49)	11.124* (1.73)	28.965*** (4.27)	18.495*** (2.78)	31.578*** (4.26)	32.140*** (4.67)	32.475*** (4.52)
South America		20.538*** (2.77)	16.311** (2.20)	29.535*** (3.61)	14.223* (1.79)	19.313** (2.53)	13.375* (1.66)	30.712*** (6.12)	20.391*** (3.06)	34.935*** (5.66)	23.828*** (3.74)	43.356*** (5.62)	43.600*** (5.85)	44.209*** (5.60)
Europe		21.414*** (3.11)	14.014** (2.13)	32.512*** (4.69)	14.990** (2.07)	23.187*** (3.17)	17.242** (2.38)	30.543*** (7.05)	17.075*** (2.75)	33.240*** (6.34)	19.511*** (2.88)	39.437*** (5.77)	39.142*** (6.01)	39.718*** (5.56)
Observations	179	179	175	110	171	141	178	155	179	144	175	91	91	91
R-squared	0.48	0.60	0.66	0.70	0.64	0.61	0.63	0.68	0.64	0.66	0.63	0.77	0.77	0.77

Method of estimation is ordinary lease squares. Robust *t* statistics are in parentheses.

***, **, *Statistical significance at the 1%, 5%, and 10% levels, respectively.

Model 1 in Table 3 shows the bivariate regression between levels of corruption, based on WGI data, and the share of people who have been vaccinated once as the dependent variable, and explains 50% of the variation in cross-country vaccination progress. In Table 4 (Model 1), corruption explains 48% of the cross-country variation in fully vaccinated people in the total population. We progressively add the regional dummies and socio-economic and health indicators to reduce the possibility of omitted variable bias. Corruption shows a robust negative association with vaccination progress in Tables 3 and 4. The negative influence of corruption is robust to controls for other socio-economic differences, as well as differences in the quality of political institutions, degree of globalization and ethnic fractionalization. Model 12 shows the effect of corruption based on WGI data on the share of people who have been vaccinated once (Table 3) and the share of people who are fully vaccinated (Table 4) after including all control variables, showing satisfactory results in line with our hypothesis. We examined the issue of multicollinearity in our general specifications and excluded two variables with *variance inflation factors* larger than 10 (government effectiveness index and domestic general government health expenditure per capita). This process has no influence on our main results.

Model 13 for both dependent variables show the net effect of the WGI corruption index on vaccination progress after considering the effects of socio-economic, health and institutional indicators. A robust negative association between cross-country vaccination progress and different levels of corruption according to both indexes can be observed in Tables 3 and 4. In Model 13 of Table 3, our measure of corruption in 2020 is from a minimum of − 2.15 (for New Zealand) to the maximum of 1.62 (for Libya). An interquartile change for this variable in Model 13 is 1.5. In this model, the share of people who have been vaccinated once ranges from 0.2% (Chad) to 82.8% (Qatar) and has a standard deviation of 26.8. The interquartile change in this variable is 51 percentage points.

In Table 3, Model 13 shows that an increase of corruption by 1.5 units (an interquartile change) is associated with a decline by 15.6 percentage points in the share of the population who have been vaccinated once, adjusting for other factors. This effect is statistically significant at 99% confidence interval, keeping other factors constant.

An increase in the (reversed) WGI corruption index measured in 2020 by 1.52 units of an interquartile change is associated with a decrease of 15.35 percentage points in the share of fully vaccinated people and can be observed in Table 4, Model 13. The effect of corruption (WGI) in this model is statistically significant at the 99% confidence interval. Among the other identified drivers of the vaccination process, the log of GDP per capita shows a positive and significant association with the share of people who are partially and fully vaccinated (Tables 3 and 4). The Polity index also shows significance in both tables, with a negative effect on the partially and fully vaccinated population. The negative association is only significant at 10% level for the fully vaccinated population. Goel et al.^18^ argue that authoritarian regimes may be more efficient in vaccination projects due to “fewer time-consuming checks and balances.” In democratic regimes, different layers of administration could also decrease trust in vaccines. In spring 2021, many European countries suspended the use of Astra Zeneca due to the risk of blood clots^61^. After a few days of suspension, the vaccine was approved by the European Medicines Agency (EMA) and its usage was resumed. However, the suspension resulted in a sharp decrease in trust in the Astra Zeneca substance^62^. Among the top countries in the list of fully vaccinated nations in our sample, we refer to countries such as Bahrain, Qatar, and United Arab Emirates with Polity index values of or near − 10 (full autocracy).

In Model 14 of Tables 3 and 4, we use the corruption index from Transparency International in 2020 as a robustness check. The level of corruption according to the CPI score has a standard deviation of 18.5 and ranges, after being re-scaled by us, from 12 (in New Zealand) to 84 (in Sudan) with an interquartile change of 30 (Table 3, Model 14). An increase of 30 units in the Transparency International public corruption index measured in 2020 in Model 14 of Table 3 is associated with a decline of 16.23 percentage points in the partially vaccinated population. This negative effect is statistically significant at 99% confidence interval. A similar increase in the Transparency International public corruption index in Model 14 of Table 4 is associated with a decline of 15.4 percentage points in the fully vaccinated population across countries and is statistically significant at a 95% confidence interval. One plausible transmission channel for the negative effect of public corruption measured by the WGI and TI indexes in 2020 on vaccinations progress is government effectiveness. In Tables 3 and 4, this dimension of governance has positive effect on vaccination progress and once it is included with corruption it reduces the significance of corruption (Models 9 in Tables 3 and 4 and Model 12 in Table 4). This shows that one of the main channels through which corruption reduces the success of vaccination projects is through its destructive effects on state capacity as is partly captured by the government effectiveness dimension of governance.

## Conclusion and policy implications

The goal of this study was to analyze the effects of public corruption on the global vaccination progress against COVID-19 across countries. We present the first cross country empirical evidence on the destructive effects of public corruption on vaccination progress. Our analysis of more than 90 countries shows that cross-country variation in corruption levels in 2020 alone explains approximately 50% of the variation in vaccination progress by the summer of 2021. An interquartile increase in the corruption index reduces the share of the vaccinated population by approximately 15 percentage points, controlling for other confounding variables. The negative effect of corruption is highly statistically significant as well. The findings also show that one of the relevant channels through which corruption negatively affected the vaccination distribution in a society is through government effectiveness. Corruption has a significant negative association with state capacity in formulating and implementing public policies and projects. The latter is a key factor for effective and efficient implementation of large-scale health projects, such as the vaccination of the greater population. Failing to control public corruption increases the vulnerability of a government’s administration and health sector in the case of pandemics like COVID-19. Corruption not only distorts economic growth but is also responsible for the lives of many people around the world by reducing the speed of vaccination and its coverage. Our empirical evidence using country-level data confirms the anecdotal evidence and reported observations in media on the destructive role of corruption in the management of the COVID-19 crisis.

Our study has important policy implications. Delay in addressing corruption, especially in the global south countries, is not only a burden for long-term economic growth but can also impose significant human capital losses during pandemics. In other words, our results show that the overall costs of corruption can be much higher when we consider its health consequences for the whole population. Corruption in government administration, which negatively affects state vaccination projects, may reduce public trust in the government and intensifies the relative deprivation of the population. Income inequality, which is higher in corrupt economies, can extend to access to health care and an increase in the share of out-of-pocket spending on health by the population. As a result, we may even expect a higher risk of conflict and violence because of corruption during the pandemic. This risk of instability is higher if the government is less capable of intervening in the market and supporting the disadvantaged population during the pandemic. There is recent evidence for the effect of COVID-19 on conflict, which is higher in countries with poor governance^63^. Our results may also hint at the possible increase in public awareness on the importance of addressing corruption. Citizens, under COVID-19, directly experience how costly public corruption can be and thus may be willing to support more anticorruption projects. Our results show that the level of economic development matters in the effective management of pandemics. Corruption, by reducing the level of economic development in the long term, may also continue to worsen health outcomes in society. One limitation of our study is that we explain cross-country variation in vaccination coverage by the summer of 2021. The pandemic is not yet resolved, and vaccinations are still on-going across the world. Therefore, future research is needed to see the full picture of the effect of corruption when the pandemic ends. Second, although we have tried to control for possible confounding variables besides our main variable of interest (corruption), one may not fully exclude the probability of missing other relevant factors. Nevertheless, we believe that this potential limitation should not be of major concern with the high explanatory power of our general specification (approximately 80%). Finally, while there are plausible arguments for the effect of corruption on the COVID-19 vaccination progress, one may also consider the possibility that these projects influence public corruption. We have tried to reduce the risk of reverse feedback by using a corruption index from 2020 and checking our results by using corruption scores from 2019. COVID-19 vaccinations intensified during 2021 across the world. It should be less likely that the COVID-19 pandemic and vaccinations programs of 2020/21 had a significant impact on corruption in 2019 or 2020. Future research may also further investigate the role of private corruption in COVID-19 vaccinations and the types of corruption practiced during the phases of vaccination development across countries.

## Supplementary Information

Supplementary Information.

## Data Availability

The datasets generated during and/or analyzed during the current study are available from the corresponding author on request. The data used for this analysis is open access. All data sources can be found in the Supplementary Information.
