# Supplementary material for: Effect of public corruption on the COVID-19 immunization progress
Source: Sci Rep. 2021 Dec 6;11:23423. doi: 10.1038/s41598-021-02802-1 (PMC8648879; doi:10.1038/s41598-021-02802-1)
Supplement: Supplementary file 1 — Supplementary Information. [file 41598_2021_2802_MOESM1_ESM.pdf]

Effect of Public Corruption on the COVID-19 Immunization Progress

Mohammad Reza Farzanegan[123*] and Hans Philipp Hofmann[1]

[1] Economics of the Middle East Research Group & School of Business and Economics, Center for Near and Middle Eastern Studies, Deutschhausstr. 12, 35037 Marburg, Germany

[2] CESifo (Munich, Germany), [3] ERF (Cairo, Egypt)

[*] email: farzanegan@uni-marburg.de

Appendix

Data description and availability

Dependent Variables	Definition	Source
Given 1dose	People vaccinated at least once with Covid-19 vaccinations as of 30.08.2021 in % of the population	Bloomberg https://www.bloomberg.com/graphics/covid-vaccine-tracker-global-distribution/
Fully vaccinated	Completed Covid-19 vaccinations as of 30.08.2021 in % of the population	Bloomberg https://www.bloomberg.com/graphics/covid-vaccine-tracker-global-distribution/
**Explanatory Variables**		
Corruption (WGI)	Control of corruption: Estimate, latest year available: 2020*-1	Worldwide Governance Indicators https://databank.worldbank.org/source/worldwide-governance-indicators
Corruption (TI)	100- Corruption perceptions index scores 2020	Transparency International https://www.transparency.org/en/cpi/2020/index/nzl
GDP per capita	Log of GDP per capita, (constant 2010 US$), average 2017-2019	World Development Indicators https://databank.worldbank.org/source/world-development-indicators
Physicians	Log of Physicians (per 1,000 people), average 2017-2019	World Development Indicators https://databank.worldbank.org/source/world-development-indicators
Government health expenditure	Log of domestic general government health expenditure per capita, PPP (current international $), average 2017-2019	World Development Indicators https://databank.worldbank.org/source/world-development-indicators
Nurses and midwives	Log of nurses and midwives (per 1,000 people), average 2017-2019	World Development Indicators https://databank.worldbank.org/source/world-development-indicators
Urban population	Urban population (% of total population), average 2017-2019	World Development Indicators https://databank.worldbank.org/source/world-development-indicators
Polity2 index	Combined Polity 2 Score in 2018 from -10 (full autocracy) to 10 (full democracy)	Marshall, M. G., Gurr, T. R. & Jaggers, K. Polity IV Project. Center for Systemic Peace (2019). Data available under the following link: https://www.systemicpeace.org/inscrdata.html
Government effectiveness	Government effectiveness: Estimate, latest year available: 2020	Worldwide Governance Indicators https://databank.worldbank.org/source/worldwide-governance-indicators
Fractionalization	Historical Index of Ethnic Fractionalization	Drazanova, L. Historical Index of Ethnic Fractionalization (HIEF). Harvard Dataverse, V2; 10.7910/DVN/4JQRCL (2019).
Globalization	KOF Globalisation Index in 2018	Gygli, S., Haelg, F., Potrafke, N. & Sturm, J.-E. The KOF Globalisation Index – Revisited. Review of International Organizations 14, 543–574; 10.1007/s11558-019-09344-2 (2019). Data available under the following link: https://kof.ethz.ch/en/forecasts-and-indicators/indicators/kof-globalisation-index.html
